# Conflict Misleads Large Carnivore Management and Conservation: Brown Bears and Wolves in Spain

**DOI:** 10.1371/journal.pone.0151541

**Published:** 2016-03-14

**Authors:** Alberto Fernández-Gil, Javier Naves, Andrés Ordiz, Mario Quevedo, Eloy Revilla, Miguel Delibes

**Affiliations:** 1 Department of Conservation Biology, Estación Biológica de Doñana, Consejo Superior de Investigaciones Científicas, Sevilla, Spain; 2 Department of Ecology and Natural Resource Management, Norwegian University of Life Sciences, Ås, Norway; 3 Departamento de Biología de Organismos y Sistemas / UMIB, Universidad de Oviedo, Oviedo, Spain; University of Lleida, SPAIN

## Abstract

Large carnivores inhabiting human-dominated landscapes often interact with people and their properties, leading to conflict scenarios that can mislead carnivore management and, ultimately, jeopardize conservation. In northwest Spain, brown bears *Ursus arctos* are strictly protected, whereas sympatric wolves *Canis lupus* are subject to lethal control. We explored ecological, economic and societal components of conflict scenarios involving large carnivores and damages to human properties. We analyzed the relation between complaints of depredations by bears and wolves on beehives and livestock, respectively, and bear and wolf abundance, livestock heads, number of culled wolves, amount of paid compensations, and media coverage. We also evaluated the efficiency of wolf culling to reduce depredations on livestock. Bear damages to beehives correlated positively to the number of female bears with cubs of the year. Complaints of wolf predation on livestock were unrelated to livestock numbers; instead, they correlated positively to the number of wild ungulates harvested during the previous season, the number of wolf packs, and to wolves culled during the previous season. Compensations for wolf complaints were fivefold higher than for bears, but media coverage of wolf damages was thirtyfold higher. Media coverage of wolf damages was unrelated to the actual costs of wolf damages, but the amount of news correlated positively to wolf culling. However, wolf culling was followed by an increase in compensated damages. Our results show that culling of the wolf population failed in its goal of reducing damages, and suggest that management decisions are at least partly mediated by press coverage. We suggest that our results provide insight to similar scenarios, where several species of large carnivores share the landscape with humans, and management may be reactive to perceived conflicts.

## Introduction

Many populations of large carnivores are threatened, usually due to anthropogenic causes [1,2,3]. This is often due to loss of habitat and high mortality levels related to depredation, other damages to properties, competition for game species, or threat to humans (e.g. [4]). On the other hand, the ongoing increase of some large carnivore populations in Europe and North America raises concern of increasing wildlife-related conflicts, as broadly defined by a confrontation between people with different views, e.g. those supporting protection and functional carnivore conservation vs. those supporters of intensive management [5,6].

Few studies on damages caused by large carnivores have actually explored the ecological, economic and societal correlates that lay behind such conflict scenarios [5,7]. However, subjective components (i.e. cultural, emotional) are important to understand and eventually mitigate wildlife-related conflicts, which may substantially affect wildlife management and conservation [8]. Furthermore, when two or more large carnivore species are sympatric, the mixture between objective (ecological, economic) and subjective components may lead to particularly complex diagnosis, as one species may suffer disproportionate negative human attitudes, unrelated to the actual magnitude of damages [7,9]. Such context calls for sound evaluation of the factors involved in conflict scenarios and the outcome of management actions [10].

Lethal population control, i.e., culling, is actually a main tool to manage large carnivores in conflict scenarios [11], implicitly assuming that carnivore abundance is a key driver of the amount of damages. Conflict scenarios related to brown bears *Ursus arctos* and wolves *Canis lupus* are common in Europe [12,13], and our study area in the Cantabrian Mountains of NW Spain is no exception [14,15]. The area holds sympatric populations of brown bears and wolves in the south-western edge of their European distributions, and both are isolated and distant from other bear and wolf populations [16]. While brown bears in Spain are listed as “critically endangered” and fully protected (about 200 individuals in the Cantabrian Mountains [17]), wolves are considered “near threatened” (about 250 packs in Spain, about 70 in the Cantabrian range [18]). Wolves are a game species in most of their Spanish range, and are also subject to regular culling. Management of bears and wolves in our study area includes economic compensations for damages. In addition, management of wolves includes annual culling programs, allegedly assuming that culling mitigates depredation on livestock and conflict.

We used records of damages to human properties and their press coverage to analyze a conflict scenario with two large carnivore species subject to distinct management. We explored correlates between damages and ecological (i.e. abundance of predators, harvested wolves, livestock numbers, harvested ungulates), monetary (economic cost of compensations) and societal (media coverage) variables. In addition, we discuss whether annual wolf culling programs followed legal mandates, and succeeded in preventing damages and reducing conflict.

## Methods

**Fig 1:**
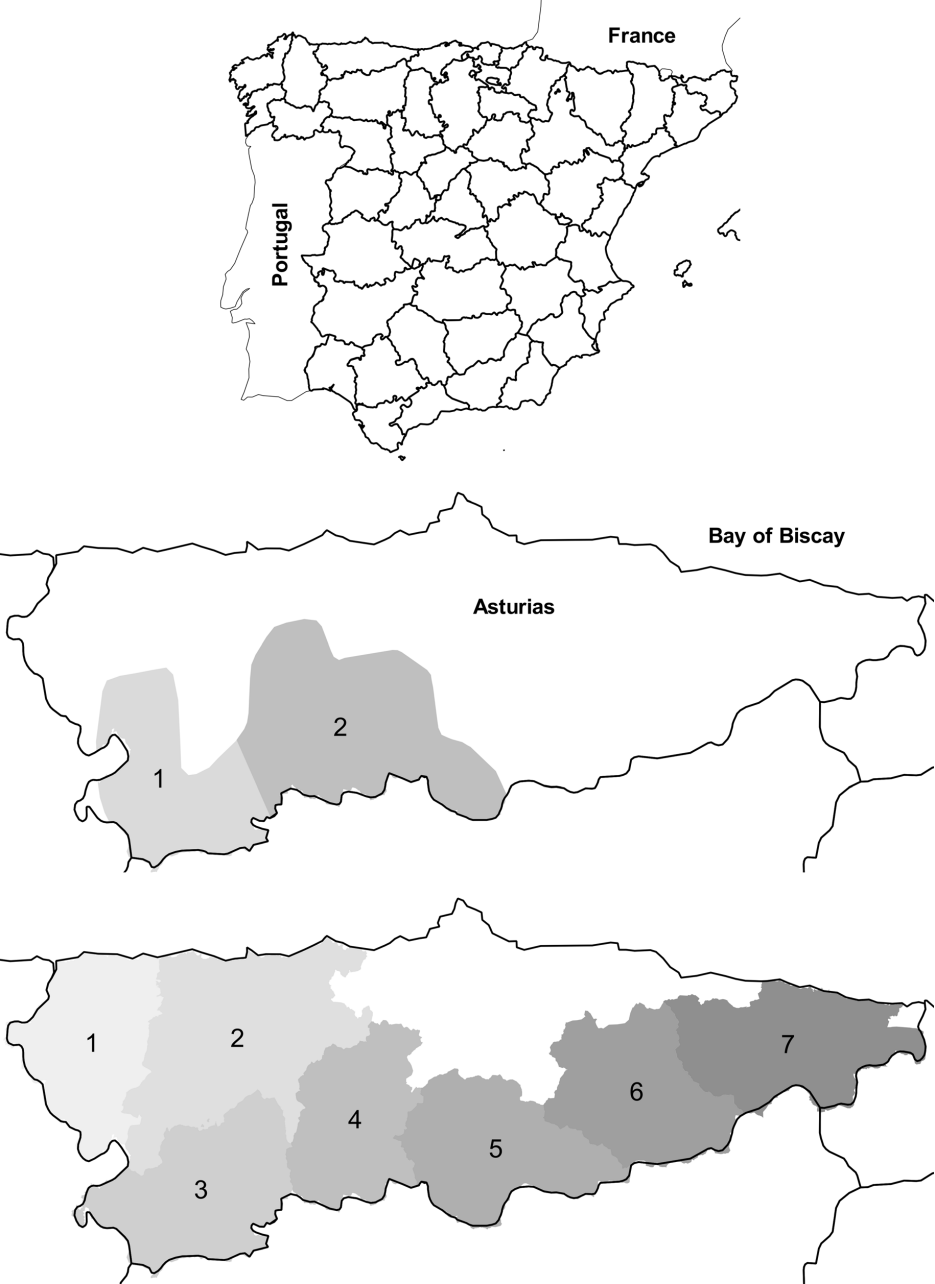
Study area. Top panel: Asturias autonomous region (NW Spain, shaded). Intermediate and bottom panels: brown bear and wolf study zones, respectively, in Asturias. Basemaps made with Natural Earth, public domain map data available at http://www.naturalearthdata.com/.

We analyzed records of complaints on depredation on beehives and livestock by bears and wolves, respectively, in the autonomous region of Asturias, NW Spain (10,604 km^2^; Fig 1). Asturias holds about 80% of the Cantabrian brown bear population [17], and about 30 packs of wolves. It is the only region in Spain that pays for damages by bears and wolves in its entire territory as part of recovery and management plans, respectively. Asturias is also the only Spanish administration that has detailed datasets of damages caused by both species. We compiled available data on wolf and bear abundance, complaints on damages by both species and details of damages, compensations paid to those complaints, livestock numbers, harvested ungulates and number of wolves killed in culling programs; all these data were provided by the regional administration with management responsibilities for both species.

Compensations of damages by bears and wolves are paid after verification by rangers in the field. Files included the number of affected beehives or livestock heads, and the amount paid as compensation in each case. Data availability was not consistent for all variables and periods; hence we used slightly different periods in the various analyses (Table 1 and S1 Dataset).

**Table 1:** Variables used in the study.

Variables	Description	Period (N years)
beehives	Response: beehives damaged by bears per year	1991–2008 (18)
depredation	Response: livestock heads depredated by wolves per year	2003–2010 (8)
Fcub	Female bears with cubs of the year in the current year	1991–2008 (18)
Fcub_-1_	Female bears with cubs of the year in the previous year	1990–2007 (18)
packs	Wolf packs in the current year	2003–2010 (8)
culled	Wolves culled in the current year	2003–2010 (8)
culled_-1_	Wolves culled in the previous year	2002–2009 (8)
ungulates_-1_^a^	Ungulates shot in the previous year	2003–2010 (8)
livestock^b^	Livestock heads (× 10^3^) per year in wolf zones	2003–2010 (8)
compensations	Annual cost of damages (€ × 10^3^) by bears and wolves	2003–2010 (8)
news	Annual news on damages by bears and wolves	2004–2010 (7)

^a^ Roe deer, red deer, wild boar and chamois hunted per year.

^b^ Sheep, goats, cattle and horses.

### Bear and wolf data

Bear management in Asturias follows a mandated recovery plan (Decree 9/2002 [19]). We used annual counts of females with cubs of the year, the only available metric of bear abundance in our study area, as a demographic surrogate for the bear population; numbers of female bears with cubs of the year were available since 1982 [20]. We differentiated two zones to analyse bear data based on well differentiated food resources [21] (Fig 1).

Bear use of anthropogenic food sources may increase when natural resources are scarce and / or when bear abundance is higher. To evaluate the latter hypothesis we used the number of damaged beehives as response variable, and the number of female bears with cubs (during any given year and in the previous one) and year as potentially explanatory variables. Claims of bear damages included beehives, livestock, orchards, and various other damages to properties. We chose the number of damaged beehives as response variable because beehives comprised 85% of damage claims to both beehives and livestock during the studied period, and 70% of monetary paid compensations; in addition, they are more robustly reported through the administrative process. The lack of reliable records on the number of beehives in Asturias prevented estimation of the proportion of beehives affected by bear attacks.

Wolf management in Asturias followed a mandated management plan during our study period (Decree 155/2002 [22]). It includes annual culling quotas of wolves based on three criteria: a) wolf abundance, b) trend and amount of damages, and c) level of social conflict. We used the official, available data on annual numbers of wolf packs, wolves killed in culling programs, attacked livestock heads and paid compensations Counts of packs were the only available annual metric of wolf abundance. There was no data available on the level of “social conflict”, or any description of its precise meaning. Data were provided by the Asturian government, the administration responsible of the wolf management plan in the whole territory of Asturias. Wolf management is divided into 7 zones; we followed a similar scheme to analyze damages on livestock (Fig 1).

Wolves in the Cantabrian Mountains prey on wild ungulates (roe deer *Capreolus capreolus*, wild boar *Sus scrofa*, red deer *Cervus elaphus* and chamois *Rupicapra parva*) and on livestock [23]. We hypothesized that livestock heads compensated for attacks by wolves per management zone and year would be positively correlated with the number of wolf packs, the number of ungulates harvested the previous year, and livestock numbers. Conversely, it would be negatively correlated with the number of wolves culled in the previous year. Data on free-ranging livestock in Asturias are publically available and updated annually [24]. Data on wild ungulates harvested by hunters per season was also provided by the regional administration.

### Media coverage of bear and wolf damages

We used media coverage as proxy of the perception of risk associated to large carnivores. Our approach is based on conceptual framework on risk judgement by the general public [25, 26], which has also been applied to perceptions of wildlife risk in mass media [27, 28]. We hypothesized that the number of damage-related news for bears and wolves would be proportional to the cost of compensations (€) paid for damages.

We searched for news on wolf and bear damages in 2004–2010 in the digital archive of the only newspaper that covers all the region of Asturias (*La Nueva España*, LNE; www.lne.es). LNE had an estimated readership of 351,000 daily readers in 2010 [29], about one third of the population of Asturias. In addition, it has three daily sub-regional editions, covering the central, eastern and western areas of the region.

To collect and classify news about damages by both species, we followed a procedure similar to [30]. Specifically, we searched for strings “oso” (bear) and “lobo” (wolf) in the digital archive of LNE. For each entry, we read first the headline of the story, which usually allowed us discarding unrelated uses of the terms (e.g. movies, surnames, etc.). Then we checked secondary headlines to allow coding stories as damages to beehives or attacks to livestock, searching also for the string “daños” (Spanish for damages, a term widely used in this context). Thereby we discriminated damage news from any other news about bears and wolves. We finally assigned each story to the municipality where it applied, and to zones in the case of wolves.

### Lethal control of wolves and management criteria

We sought to determine if the number of wolves legally killed every year in each zone was related to wolf management criteria: a) the number of wolf packs present per year and zone; b) compensations paid (€) for verified damages per year and zone; and c) the number of damage-related news per year and zone, as a proxy to conflict. The analysis of media coverage of wolf damages per zone was restricted to 2006–2009, when media archives allowed assigning news to specific zones.

### Data analysis

First, we analysed if there were trends in the variables (exponential growth rate), fitting generalized linear models (GLM; Poisson distribution) with year as explanatory variable. Then we fitted generalized mixed models (GLMMs with negative binomial distribution, logit link function) [31] to damages, with zone as random factor. We evaluated model performance and parsimony using Akaike Information Criteria (AIC), the difference (ΔAIC) between each candidate model and the best model (lowest AIC), and AIC weights (AICw [32]). Analyses were performed in R and SAS [33, 34].

## Results

In the study area there were 8 ± 3 female bears with cubs per year (mean ± SD). Bears damaged 250 ± 237 beehives annually, and the cost of bear damages averaged 127,203 ± 39,779 € per year. The three variables increased over the study period (Table 2). News on bear damages amounted to just 3 ± 1.3 per year (mean ± SD), preventing trend analysis. Beehives damaged by bears in any given year and zone were positively related to the number of bear females with cubs in the previous year (Table 3).

**Table 2:** Trends in the variables used in the study.

Variables	EGR^a^ (± SE)	P
beehives	0.19 ± 0.03	< 0.001
depredation	0.05 ± 0.01	< 0.001
Fcub	0.06 ± 0.01	< 0.001
packs	0.01 ± 0.03	NS
culled	0.03 ± 0.06	NS
ungulates	0.04 ± 0.01	< 0.001
livestock	-0.02 ± 0.01	< 0.001
compensations (bears)	0.09 ± 0.03	0.01
compensations (wolves)	0.10 ± 0.01	< 0.001
news (bears)	0.05 ± 0.11	NS
news (wolves)	-0.12 ± 0.02	<0.001

^a^ Annual trend of each variable estimated as exponential growth rate (± SE) via GLMs with Poisson distribution.

**Table 3:** Models fitted to beehives damaged by bears, and to livestock heads depredated by wolves.

**beehives**^**b**^	**AIC**	**ΔAIC**	**AICw**	**ß ± SE**^**a**^	**P**
null model	411.5	17.3	0		
Fcub + Fcub_-1_ + year	395.7	1.5	0.32		
Fcub_-1_ + year	394.2	0	0.68		
**Variables retained**					
Fcub_-1_				0.27 ± 0.12	0.03
year				0.14 ± 0.04	0.002
**depredation**^c^					
null model	733.1	25	0		
packs+culled+culled_-1_ +ungulates_-1_ +livestock	711.1	3	0.13		
packs +culled +culled_-1_ +ungulates_-1_	709.5	1.4	0.29		
packs +culled +culled_-1_	708.1	0	0.58		
**Variables retained**					
packs				0.06 ± 0.03	0.08
culled				0.09 ± 0.02	0.001
culled_-1_				0.07 ± 0.02	0.001

GLMM models with negative binomial distribution and zone as random factor. AIC is Akaike Information Criterion; ΔAIC is the difference between best model (lowest AIC) and each candidate model; AIC_w_ are AIC weights.

^a^ Estimate and standard error for the variables retained in the best models.

^b^N = 36; 18 years, two zones.

^c^N = 56; 8 years, 7 zones.

Variables: Fcub, number of bear females with cubs of the year; Fcub_-1_, number of bear females with cubs of the year in the previous year; packs, number of wolf packs in the current year; culled, number of wolves killed in the current year; culled_-1_, number of wolves killed in the previous year; ungulates_-1_, number of ungulates shot in the previous year; livestock, heads of livestock present in the current year.

In the study area and period there were 29 ± 5 wolf packs per year (mean ± SD). 15 ± 7 wolves per year were killed in culling programs. The annual number of livestock heads affected by wolf damages averaged 2,951 ± 478, and increased during the study period (Table 2). Compensation costs of wolf damages averaged annually 691,498 ± 201,687 €, and also increased during the study period (Table 2). Livestock heads compensated by depredations amounted to 0.69 ± 0.14% of free-ranging livestock, which averaged 423,079 ± 29,136 heads per year in the study area.

**Fig 2:**
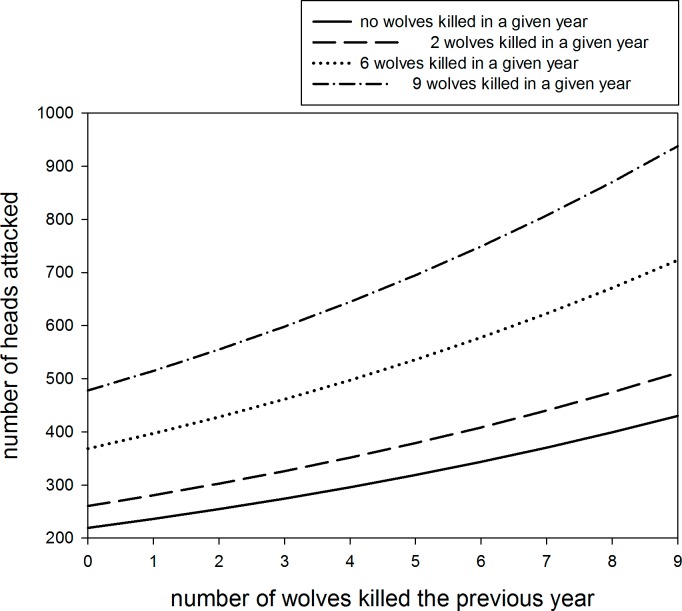
Relationship between the number of livestock heads depredated by wolves and number of wolves culled the previous year. The plot is based on the best model of wolf depredation on livestock; the model was parameterized for different numbers of wolves killed in the current year, and in a zone harboring the average number of packs per zone (N = 4).

Livestock depredation in any given year and zone was positively related to wolf packs and the number of wolves culled both during the current and the previous year (Table 3; Fig 2). The second and third best models also retained a positive effect of the number of ungulates harvested in the previous year (Table 3). 70% of compensated livestock heads (N = 13,194) were lost between April and October. 7,976 ± 1,011 wild ungulates were shot per year in the study area.

Overall, media coverage on wolves and bears was similar (125 ± 32 and 116 ± 29 news per year, respectively; mean ± SD). The cost per complaint averaged 339 € for wolves and 505 € for bears, although total compensations paid were five times higher for wolves than for bears. The total number of news on wolf damages was 30 times higher than news on bear damages. Media coverage on wolf damages per zone was also uncorrelated to the economic cost of damages (Kendall’s tau correlation coefficient = 0.17; N = 35; five years, seven zones).

**Fig 3:**
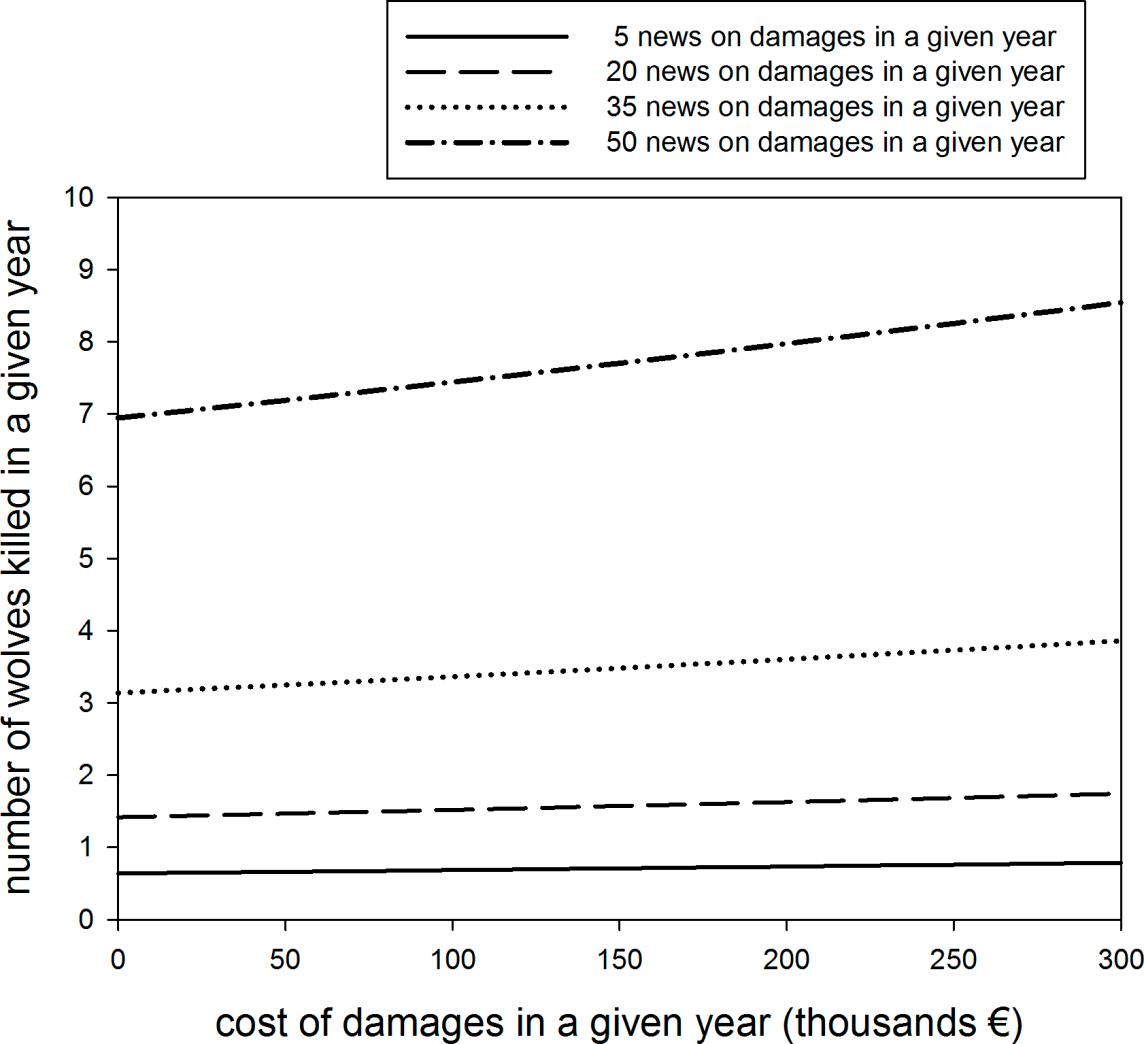
Relationship between wolves culled and compensated damages. The plot is based on the best model relating wolves culled in a given year and the cost of damages compensated in that year, as a function of the number of news on damages published in that year.

Most wolves were killed between January and August (71%; N = 101), i.e. including the wolf breeding season. The annual number of wolves culled in each zone ranged from 0 to 11, with an average of 2 individuals per zone and year. Wolf culling was positively related to the number of news on wolf damages per zone, and to paid compensations (Table 4; Fig 3). The number of packs per zone (average = 4; range 1–8) was also retained in the second best model (Table 4).

**Table 4:** Models fitted to the number of wolves culled per year.

	AIC	ΔAIC	AICw	ß ± SE^a^	P
null	110.7	9.7	0		
packs + compensations + news	102.7	1.7	0.30		
compensations + news	101	0	0.70		
**Variables retained**					
compensations				0.001 ± 0.0002	0.006
news				0.053 **±** 0.018	0.008

GLMM models with negative binomial distribution and zone as random factor; N = 28 (four years, seven zones). AIC is Akaike Information Criterion; ΔAIC is the difference between best model (lowest AIC) and each candidate model; AIC_w_ are AIC weights.

^a^ Estimate and standard error for the variables retained in the best model. Variables: packs, number of wolf packs; compensations: cost of complaints due to livestock depredation by wolves (€); news: number of news published on livestock damages by wolves.

## Discussion

Conflict scenarios rooted in human attitudes and confronting perceptions of large carnivores, e.g. groups that oppose carnivore recovery vs. carnivore supporters, are major obstacles for carnivore conservation and recovery [35]. Therefore, disentangling the relative importance of ecological, economic and societal factors involved in human-carnivore interactions should facilitate coexistence [36]. We used the number of news on wolf damages per zone as a proxy of social conflict, and found that the press coverage of wolf damages was not correlated to their economic costs. The unbalanced press coverage is relevant because news stories on damages correlated to wolves killed in management actions (Fig 3; Table 4). Media coverage is thus a potential driver of public risk perception of large carnivores (e.g. [26, 28]), showing that conflict resolution does not necessarily lay just on ecological grounds [37], or in science communication. Indeed, social factors may influence management actions (e.g. Fig 3).

We found that livestock damages were positively correlated to wolf culling intensity in the previous year, hinting an undesired outcome of management based on culling. The relation between wolf culling and subsequent damages corresponded to a set of paired years and wolf zones (Fig 2; Table 3); it did not depend on overall trends in wolf numbers or damages, but actually showed a relation between culling and the number of damages the year after. Previous studies showed that culling or hunting do not necessarily minimize depredation on livestock [38,39] and recent research in North America even found similar counter-expected effects in black bears, pumas, and wolves [40,41,42]. To our knowledge, a positive correlation between number of culled large carnivores and increased damages has never been published in Eurasia. Several plausible scenarios could explain those effects: source-sink hypothesis (e.g. [41]), and social disruption, i.e., an outcome of random culling in highly social animals like wolves [43]. Culling reduces pack size, which together with the social disruption caused by killing reproductive individuals could result in an increase of the number of packs in a region [44, 45]. In addition, kill rates in wolves depend on season, pack size, prey size and prey density, among others [46, 47]. Kill rates seem to be higher in Europe than in North America, perhaps indicating that higher risk of human-related mortality in European wolves leads to a decline in consumption of each carcass [47, 48]. Although the levels of damages on livestock in our study area may seem disparate for the number of packs and average pack size [49], the observed pattern could arise if wolves spent less time at kills because livestock owners and rangers visit the carcasses. A similar effect has been described for pumas living closer to human residential areas [50].

Availability of wild prey is also an important factor behind carnivore predation on livestock [51, 52]; abundant wild prey may avert predation on livestock. However, data are rarely available to test that idea [53]. We did not have robust data on abundance of wild prey, but our surrogate (ungulates harvested in the previous season) showed a positive correlation with the number of damages by wolves on livestock. Furthermore, unguarded livestock is susceptible to depredation even if wild prey is available [54], adding a human-dependent issue to predator-prey interactions. Livestock husbandry is an objective component that plays a major role in the magnitude of damages by large carnivores [55, 56]. Yet, hard data on type and dedication of husbandry practices are absent in our study area.

The number of bears in the Cantabrian Mountains increased during the study period, coinciding with an increase in damages to beehives. A simple explanation would be that bears shift to anthropogenic resources when the natural ones are scarce, thus increasing damages to human properties. However, we found that bear damages correlated with females with cubs in the previous year. This may indicate that an increase in the proportion of juvenile bears in the population–which have faster growth rates and are often less wary—lead to an increase in damages to beehives.

Bear damages did not seem as conflictive to the press as wolf damages, judging from the dramatic skew in the treatment of damages by bears and wolves: compensations paid annually for wolf damages were indeed five times higher than those paid for bear damages (691,498 v. 127,203 € per year), yet media coverage of wolf damages was 30 times larger (91 v. 3 news per year). Such bias and its potential effects on management can remain undetected when studying only one of several sympatric species in a conflict scenario [12, 57].

### Management and conservation implications

A widespread measure to increase social acceptance of large carnivores is to compensate economically the damages they caused [11, 58]. In our study area, about 85% of the complaints were compensated after verification, but compensations did not seem to ease conflict. It is worth noting that stockbreeding activities are subsidized by the Common Agricultural Policy (CAP) of the European Union. Those subsidies are higher for livestock grazing in protected areas, to compensate restrictions associated to them, including potential inconveniences of sharing the landscape with large carnivores and wild ungulates [59, 60].

The situation we described urges the implementation of better livestock husbandry practices instead of wolf culling, which is counterproductive from damage-management and conservation perspectives. Indeed, improving livestock handling is often regarded as the most rational and conservation-oriented measure in different scenarios. It also calls for attention to the role of media and opinion makers as potential amplifiers or drivers of wildlife-related conflicts: wolf depredation affected annually 0.69 ± 0.14% of free-ranging livestock in our study area, i.e., depredation is not a major cause of livestock mortality, but media is seemingly driving the implementation of culling programs.

Culling of populations of apex predators is unjustified on scientific grounds [61]; indeed, culling suppress certain ‘apex’ traits [62, 63], thus altering their role in ecosystems. In addition, the implementation and outcome of conflict-related management actions on large carnivores should also be evaluated on ethical grounds [45, 64].

## Supporting Information

S1 Dataset Data on damages by bears and wolves used in the analyses. Data on bear and wolf damages, numbers of female bears with cubs, wolf packs, wolves killed in culling programs, harvested wild ungulates, and news on wolf damages used in the analyses of this study. See Table 1 for description of variables.
(XLS)
